# Development and Validation of an Osteoporosis Self-Assessment Tool for Taiwan (OSTAi) Postmenopausal Women-A Sub-Study of the Taiwan OsteoPorosis Survey (TOPS)

**DOI:** 10.1371/journal.pone.0130716

**Published:** 2015-06-18

**Authors:** Fu-Mei Su, Dung-Huan Liu, Jia-Feng Chen, Shan-Fu Yu, Wen-Chan Chiu, Chung-Yuan Hsu, Chi-Hua Ko, Ching-Chou Tsai, Tien-Tsai Cheng

**Affiliations:** 1 Division of Rheumatology, Allergy, and Immunology, Department of Internal Medicine, Kaohsiung Chang Gung Memorial Hospital, Kaohsiung, Taiwan; 2 Department of Physical Medicine and Rehabilitation, Taipei Tzu Chi Hospital, Buddhist Tzu Chi Medical Foundation, New Taipei City, Taiwan; 3 Chang Gung University College of Medicine, Kaohsiung, Taiwan; 4 Department of Obstetrics and Gynecology, Kaohsiung Chang Gung Memorial Hospital, Kaohsiung, Taiwan; University of Zaragoza, SPAIN

## Abstract

**Background:**

To develop an OSTAi tool and compare this with the National Osteoporosis Foundation recommendations in 2013 (NOF 2013) for bone mineral density (BMD) testing among Taiwan postmenopausal women.

**Methods:**

Taiwan Osteoporosis Association (TOA) conducted a nationwide BMD survey by a bus installed with a dual energy X-ray absorptiometry (DXA) between 2008 and 2011. All of the participants completed questionnaire, which included demographics and risk factors of osteoporotic fracture in FRAX tool. We used the database to analyze potential risk factors for osteoporosis and followed the model by Koh et al. to develop a risk index via multiple variable regression analysis and item reduction. We used the index values to set up a simple algorithm (namely OSTAi) to identify those who need BMD measurement. Receiver operating characteristic (ROC) curve and the area under the curve (AUC) was used to compare the sensitivity/specificity analysis of this model with that of recommendations by NOF 2013.

**Results:**

A total of 12,175 Taiwan postmenopausal women enrolled in this survey. The index value was derived by age and body weight of the participants according to weighted odds of each risk factor and the selected cutoff value was set at “-1”. There are 6393 (52.5%) participants whose index value is below “-1” and whose risk of osteoporosis was 57.5% (3674/6393). The AUC for OSTAi and NOF 2013 were 0.739 (95% confidence interval (CI), 0.728–0.749, P<0.001) and 0.618 (95% CI, 0.606–0.630, P<0.001), respectively. The sensitivity and specificity of OSTAi, at the selected cutoff value of -1, and NOF 2013 to identify osteoporosis were 73.1%, 62.0% and 78.3%, 45.7%, respectively.

**Conclusions:**

As OSTA for Asian populations, OSTAi is an useful tool to identify Taiwan postmenopausal women with osteoporosis, In comparison with NOF 2013, OSTAi may be an easier and better tool for referral to BMD measurement by DXA in this area.

## Introduction

Osteoporosis is a systemic skeletal disorder characterized by low bone mass and microarchitectural deterioration of bone tissue, and as a consequence, an increase in bone fragility and susceptibility to fracture [1].

Several previous studies concluded that advanced age and low body weight are strongly associated with osteoporosis and with increased fracture risk [2–5]. As life expectancy increased, osteoporosis has become a major public health problem worldwide. Taiwan is now an aging society and is expected to become an aged country (considered to be when the elderly population exceeds 14% of the total population) in 2017 [6]. Thus, identification of women at risk for osteoporosis is extremely important for the prevention of osteoporotic fractures and reducing economic burden in Taiwan.

BMD assessment by DXA is the standard test to diagnose osteoporosis (T-score ≤ -2.5), according to the classification of the World Health Organization (WHO) criteria [7]. However, routine DXA scan for all subjects is not practical in Taiwan because of limitation of reimbursement policy or unavailability of the DXA facilities in some rural areas.

Several tools and recommendations [8–17] have been developed to select subjects at increased risk of osteoporosis for further BMD measurement. The OSTA, developed by Koh and colleagues in 2001[9], is a tool to assess the risk of osteoporosis simply based on age and weight of the subjects. Subsequently, the model developed by Koh et al. has been validated and reported to be an easy and effective assessment tool in several diverse populations [18–23].

Nevertheless, the performance of OSTA has not yet been directly evaluated in large samples of Taiwan postmenopausal women. In addition, a survey for the incidence rate of hip fracture from 1996 to 2000 in Taiwan revealed a high annual incidence rate of hip fracture for both men and women [24]. Moreover, the types and distributions of risk factors for osteoporosis in Taiwan postmenopausal women may potentially differ from those among other Asian countries. Therefore, we follow the original OSTA model with some adjustment by altering the risk category to develop a simple screening tool, namely OSTAi, for assessing the risk of osteoporosis in Taiwan postmenopausal women.

The NOF 2013 was developed to select individuals who should undergo DXA testing. As a screening tool, it is uncertain whether NOF 2013 performs as well as other models. The OSTA model had been validated in several countries and populations with different method [14, 25–29]. However, to our knowledge, the model had not been validated by comparison with NOF 2013 in published literature. Thereby, we compared the performance of OSTAi with NOF 2013 in predicting osteoporosis.

## Materials and Methods

### Participants

TOA conducted a nationwide circuit program for BMD measurements between 2008 and 2011. This program, sponsored by Merck Sharp & Dohme pharmaceutical company, made use of a bus installed with a DXA machine (Explorer; Hologic Inc., Waltham, MA), a well trained nurse, and an International Society of Clinical Densitometry (ISCD) certified radiology technician. The bus circulated to regions or hospitals on request for BMD measurement. Participants, including pre- and postmenopausal women and men, were recruited consecutively from 104 sites located countrywide in Taiwan during the period.

The participants enrolled in current investigation must fulfill the inclusion criteria that included Taiwanese women, postmenopausal for ≥12 months, hip anatomy suitable for evaluation by DXA scans, willingness to participate in the study, and ability to read and provide informed consent. As this project was a screen program, no specific exclusion criteria had been set. However, those subjects who could not access the machine or had had both hips previously fractured or replaced were excluded.

### Ethics statement

This study was approved by the local Institutional Review Board of Chang Gung Memorial Hospital (102-1878B). All participants provided their written informed consent in this study.

### Data collection and Measurements

Each participant was interviewed by a well-trained nurse to complete the questionnaire that incorporated risk factors of osteoporotic fracture in the FRAX tool. These variables included age, gender, body height (BH), body weight (BW), previous fragility fracture, parent fractured hip, premature menopause, current smoking, glucocorticoids use, rheumatoid arthritis, secondary osteoporosis, and alcohol consumption.

The BW, to the nearest 0.1 kilogram (kg) and BH, to the nearest 0.1 centimeter (cm) of each participant were measured using a calibrated spring balance with a ruler, with the subject wearing light clothing without shoes.

All BMD measurements, including lumbar spine and hip regions, were performed by the DXA machine in the bus. The BMD values, expressed in g/cm2, were converted into T scores, expressed in standard deviations (SDs), based on the National Health and Nutrition Examination Survey (NHANES III) reference values for women aged 20–29 years, which is the recommended reference database for Taiwanese population [30, 31].

The participants were classified as non-osteoporotic or osteoporotic risk group with the need of BMD testing according to whether they fulfill one of the following criteria from the NOF 2013 recommendations. The criteria included as below: women age 65 and older, or postmenopausal women younger than age 65 with one or more clinical risk factors for fracture such as low body weight (BMI < 18.5 kg/m ^2^), prior fracture, high risk medication use associated with low bone mass or bone loss (e.g., glucocorticoid in a daily doses ≥ 5 mg prednisone or equivalent for ≥ three months), or disease or condition associated with bone loss (e.g., rheumatoid arthritis). The diagnostic performance of NOF 2013 for predicting osteoporosis in our studied population was then assessed.

### Statistical Analysis

We followed the statistical analysis by Koh et al. with some modification. The potential risk factors for osteoporosis included in the model development process consisted of the risk factors in the FRAX tool, in which ethnic factor was not included.

Analysis was performed in two stages: first to identify risk factors with mutually independent associations, and then to develop a simple index from these variables, reducing the number of variables to as few as possible while retaining good performance.

At first, univariate models were performed, using BMD T-score as the dependent variable, and each risk factor of osteoporotic fracture in the FRAX tool as the independent predictor. Only statistically significant (p<0.05) variables were considered as major risk factors and remained in a multiple variable regression model. Next, weighting and then combining the index weight values of major risk factors were used to constitute the OSTAi index through multiple variable regression analysis and item reduction method.

Osteoporosis is defined as T-score at any site (lumbar spine, femoral neck, or total hip) is ≤ − 2.5, according to the WHO criteria. The ability of OSTAi to discriminate each participant with osteoporosis was evaluated by ROC curve analysis. The sensitivity was defined as the proportion of women with osteoporosis (T-scores ≤− 2.5) that tested positive (i.e., index values below the cutoff) and specificity was defined as the proportion of women without osteoporosis who tested normal (i.e., having index values above or equal to the cutoff). The AUC was used to compare the diagnostic performance of OSTAi with that of NOF 2013.

In this study, the data are expressed as mean ± SD. Continuous variables were evaluated by t-test. Categorical variables were evaluated by Chi-square test. All analyses were performed using the Statistical Package of Social Science for Windows software version 17 (IBM SPSS Statistics 17). A p value less than 0.05 was considered statistically significant.

## Results

**Fig 1:**
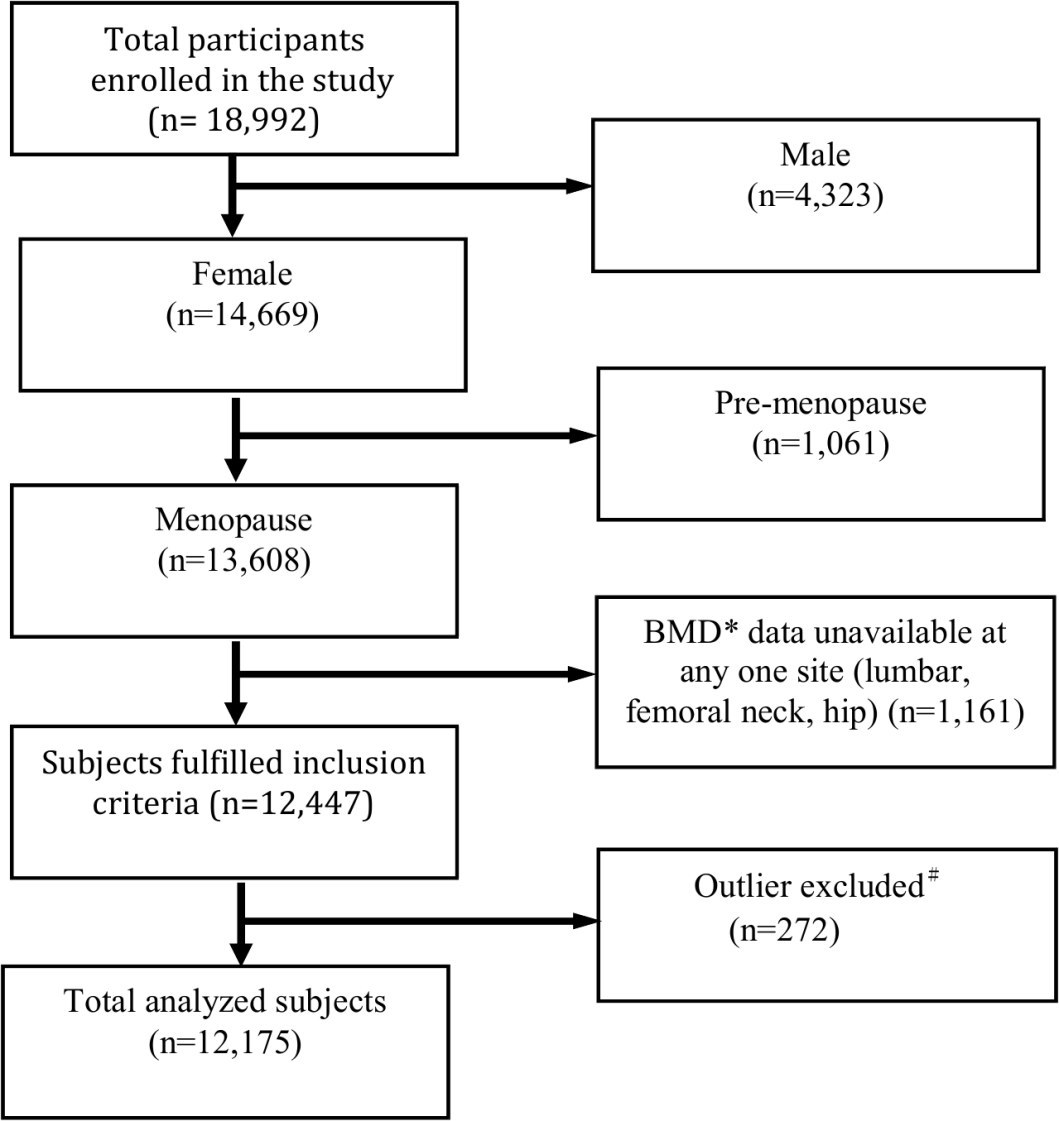
Disposition of participants. Participants whose BMD was missing at any one site for any reason and participants with extreme values (deviating from the mean by more than three times the standard deviation) were excluded from data analysis.

A total of 18,992 participants, including 4,323 (22.8%) male and 14,669 (77.2%) female, were enrolled for BMD measurement between 2008 and 2011 in this program. Those who were male, premenopausal women and BMD data unavailable at any one site (lumbar spine, femoral neck, and total hip) were excluded from analysis. Participants with extreme values (deviating from the mean by more than three times the standard deviation), including demographics (age, height, weight, age of menopause) were also excluded from data analysis. The disposition of participants is depicted in Fig 1. A total of 12,175 Taiwanese postmenopausal women were analyzed.

Selected characteristics of the studied population are presented (Table 1). The average age was 66.0 ± 9.6 years. The mean BW and mean body mass index (BMI) were 56.9 ± 8.8 kg and 24.0 ± 3.5 kg/m^2^, respectively. The prevalence of osteoporosis (T-score ≤ −2.5 at lumbar spine, femoral neck, or total hip) of our cohort (n = 12,175) was 41.3%. Whereas, the proportions of participants with T-scores ≤ −2.5 at lumbar spine, femoral neck, and total hip was 32.7%, 25.3%, 8.8%, respectively.

**Table 1:** Demographics of participants.

Characteristics	Total N = 12175 (100%)	T-score# > -2.5 N = 7148(58.7%)	T-score# ≤ -2.5 N = 5027(41.3%)	P value
Age (years)	66.0±9.6	63.3±8.9	69.8±9.4	<0.001
Height (cm)	153.9±5.8	155.1±5.4	152.1±5.8	<0.001
Weight (kg)	56.9±8.8	59.0±8.5	53.9±8.5	<0.001
Body mass index (kg/m^2^)	24.0±3.5	24.5±3.4	23.3±3.5	<0.001
Age of menopause (years)	49.3±4.5	49.5±4.5	49.0±4.5	<0.001
BMD (T-score)				
Lumbar spine	-1.8±1.3	-1.1±1.0	-2.8±0.8	<0.001
Femoral neck	-1.8±1.1	-1.2±0.8	-2.6±0.8	<0.001
Total hip	-1.0±1.1	-0.4±0.9	-1.8±0.9	<0.001
Other risk factors*in FRAX		n/N^@^(%)		
Parent Fractured Hip	840/7826(10.7)	501/4630(10.8)	339/3196(10.6)	0.795
Previous Fracture	1294/8624(15.0)	529/4954(10.7)	765/3670(20.8)	<0.001
Glucocorticoids	576/8507(6.8)	313/4918(6.4)	263/3589(7.3)	0.088
Rheumatoid arthritis	631/8494(7.4)	365/4891(7.5)	266/3603(7.4)	0.900
Secondary osteoporosis	727/8617(8.4)	438/4951(8.8)	289/3666(7.9)	0.117
Current smoking	111/8730(1.3)	56/5013(1.1)	55/3717(1.5)	0.147
Alcohol 3 or more units/day	59/8671(0.7)	34/4978(0.7)	25/3693(0.7)	0.973

# Lowest T-score among lumbar spine, femoral neck and total hip

* Definition same as those of FRAX

@ n = answered yes in the questionnaire,

N = total number who answered the questionnaire

The absolute number and the proportion of women whose T-score was at −2.5 or below at the lumbar spine, the femoral neck, the total hip, or a combination of two or all three sites are shown in Table 2. Of the 5,027 women with diagnosis of osteoporosis, T-scores of −2.5 or below were found in 3,985 at the lumbar spine, in 3,084 at the femoral neck, and in 1,072 at the total hip. These figures correspond to the total number of participants with T-scores at −2.5 or below at each site. For example, the figure of 3,985 for the lumbar spine includes 1,896 women with T-score at −2.5 or below at the lumbar spine only, plus 1,209 women with T-score at −2.5 or below at both lumbar spine and femoral neck, plus 31 women with T-score at −2.5 or below at both lumbar spine and total hip, plus 849 women with T-score at −2.5 or below at lumbar spine, femoral neck and total hip.

**Table 2:** Number of woman with T-score at -2.5 or below according to site.

Site(s)with a T-score ≦-2.5	N	Percent (%)
Lumbar spine only	1896	37.7
Femoral neck only	850	16.9
Total hip only	16	0.3
Lumbar spine + femoral neck	1209	24.1
Lumbar spine +total hip	31	0.6
Femoral neck +total hip	176	3.5
Lumbar spine + femoral neck + total hip	849	16.9
Total	5027	100

The univariate analyses revealed that seven factors including age, BH, BW, menopausal age, previous fracture, current smoking, and glucocorticoids use were major risk factors of osteoporosis. Weighting of these seven variables which derived from multiple variable regression model were then used to calculate the OSTAi index value for each participant. The regression coefficient and standard error for each variable, along with the index weights, are shown in Table 3.

**Table 3:** Regression coefficients for the univariate and multiple variable model.

	Univariate analysis			Multivariable analysis			
Variable	β	SE*	P	β	SE*	P	Index weight
Age (*vs*.10 years younger)	-0.433	0.009	<0.001	-0.364	0.011	<0.001	-2
Height (*vs*.10 cm shorter)	0.573	0.016	<0.001	0.175	0.019	<0.001	1
Weight (*vs*.10 kgs lighter)	0.439	0.010	<0.001	0.343	0.012	<0.001	2
Postmenopausal age (*vs*.10 years younger)	0.145	0.021	<0.001	0.161	0.022	<0.001	1
Previous fracture (*vs*. no)	-0.464	0.032	<0.001	-0.228	0.028	<0.001	-1
Smoking (*vs*. no)	-0.227	0.102	0.026	-0.293-	0.090	0.001	-2
Glucocorticoids (*vs*. no)	-0.122	0.046	0.008	-0.198-	0.039	<0.001	-1
Parent hip fracture (*vs*. no)	0.018	0.039	0.634	-	-	-	
Rheumatoid arthritis (*vs*. no)	-0.026	0.044	0.552	-	-	-	
Secondary osteoporosis (*vs*.no)	0.064	0.041	0.124	-	-	-	
Alcohol (*vs*. no)	-0.002	0.140	0.987	-	-	-	

*SE: standard error

**Fig 2:**
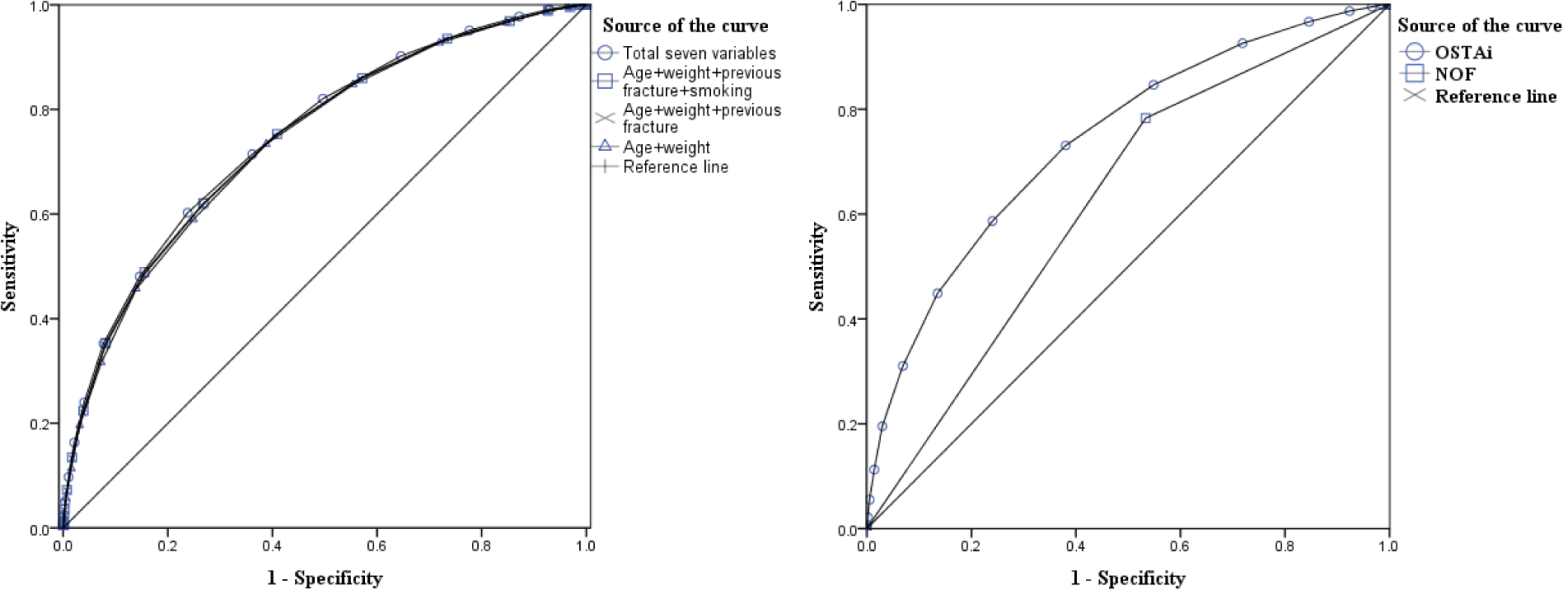
The ROC curves for comparison. (a)The ROC curves for final model variables. ROC curves of two-variable model (age and weight), three-variable model, four-variable model, and total seven variables in predicting osteoporosis (b)The ROC curves for the OSTAi index and NOF 2013 recommendations.

The ROC curve analysis is displayed in Fig 2(A). The AUC for the model based on all seven variables was 0.749 (95% CI, 0.738–0.759, P<0.001). The AUC for the model based on age and BW was 0.739 (95% CI, 0.728–0.749, P<0.001). By item reduction step by step, it revealed that if only age and BW remained in the model, the performance of discrimination was similar to that of models with seven variables. Thereby, the risk index (namely OSTAi), based on age and BW, could be obtained by adding -2 units per ten years increase in age and 2 units per 10 kgs increase in BW to the referent age (50-year-old) and BW (50kgs). This finally equals to subtract age in years from weight in kg, multiply by 0.2, and round off the result to an integer. The OSTAi value was calculated as 0.2 (weight in kg–age in years) and then rounded off to an integer. OSTAi index values of 12,175 participants were calculated accordingly. The median, the mean, SD, and range of OSTAi index values were -2, -1.82, 2.77, and -12 to 8, respectively.

The optimal cutoff of OSTAi index values to identify those subjects with osteoporosis was estimated by ROC curve. Integer -1 was selected as the best cutoff, which yielded best sensitivity (73.1%) and specificity (62.0%)evaluation in our cohort. The positive predictive value (PPV) was 57.5%, and the negative predictive value (NPV) was 76.6% (Table 4), respectively. While the sensitivity and specificity of NOF 2013 were 78.3% (3936/5027) and 46.7% (3335/7148). The PPV, NPV, and AUC of NOF 2013 were 50.8% (3936/7749), 75.4% (3335/4426), and 0.618 (95% CI:0.606–0.630, P<0.001), respectively (Table 4). The ROC curves for the OSTAi index and NOF 2013 are displayed in Fig 2(B). The AUC for OSTAi and for NOF 2013 were 0.739 (95% CI: 0.728–0.749, P<0.001) and 0.618(95% CI: 0.606–0.630, P<0.001), respectively. The performance of OSTAi and NOF 2013 in predicting osteoporosis was compared by the ROC curves.

**Table 4:** Positive and negative predictive value of OSTAi index and NOF 2013.

	Osteoporosis^a^		
	(-)	(+)	
	n		N
OSTAi index value<-1			
yes	2719	3674 (57.5 ^b^, 73.1^c^)	6393
no	4429 (76.6 ^d^, 62.0^e^)	1353	5782
NOF 2013			
yes	3813	3936 (50.8 ^b^ ^,^ 78.3^c^)	7749
no	3335 (75.4 ^d^, 46.7^e^ ^)^	1091	4426

a, T-score ≤-2.5 at femoral neck, total hip, or lumbar spine;

b, Positive predictive value;

c, Sensitivity;

d, Negative predictive value;

e, Specificity

**Fig 3:**
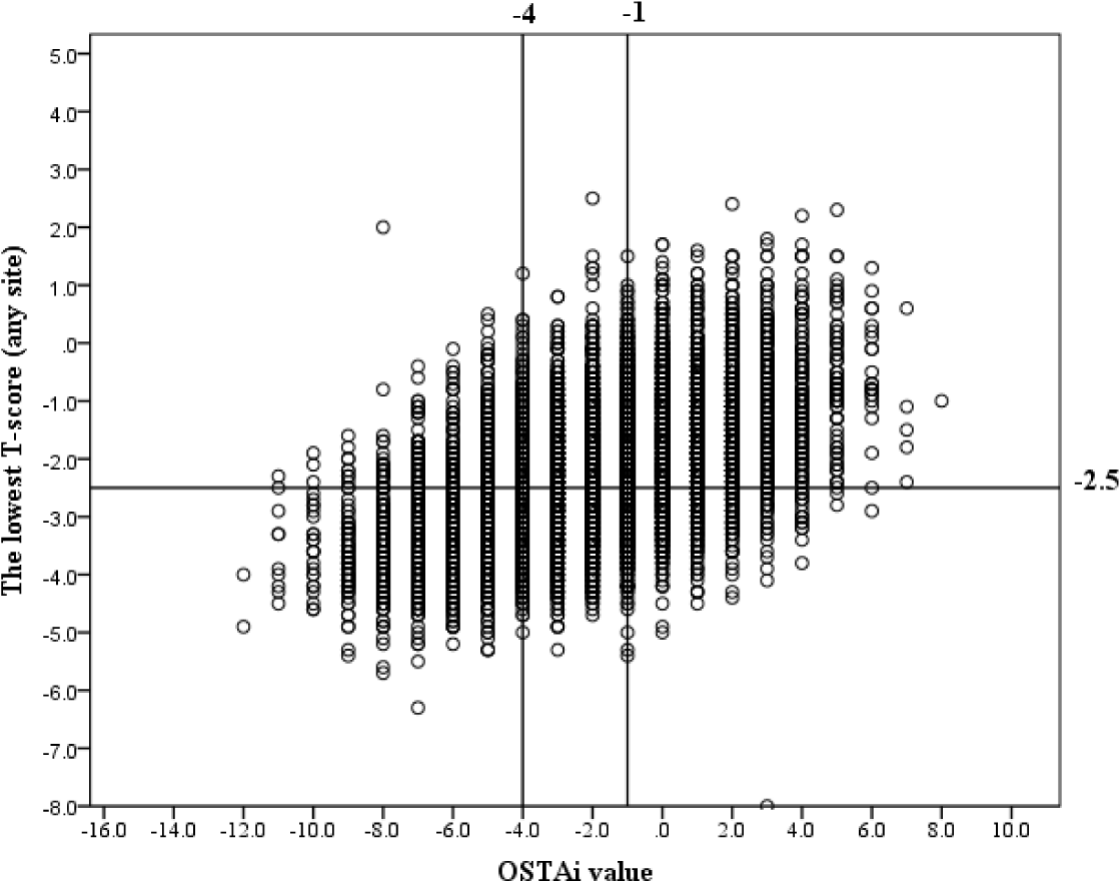
A plot of the OSTAi value versus the lowest T-score at any site. The horizontal line indicates a T-score of -2.5. The vertical lines mark the OSTAi value cutoffs of -1 and -4, which identified postmenopausal women at low (≥ -1), medium (-1 to -4), and high risk (≤-4) for osteoporosis.

Based on the osteoporosis risk categories used in the Koh study [9], three risk categories were arbitrarily created using the OSTAi index value with cutoffs of -1 and -4. OSTAi values versus lowest T-scores for the entire sample was displayed in Fig 3. In current investigation, participants with OSTAi index values ≤-4 were classified as the high-risk group, those with OSTAi index values between -1 and -4 (OSTAi <-1 and >-4) were classified as the medium-risk group, and those with OSTAi index values ≥-1 were classified as the low-risk group. In the present sample 47.5%, (5782/12175) of women were in the low-risk category, 26.0% (3170/12175) in the medium-risk category, and 26.5% (3223/12175) in the high-risk category. The prevalence of osteoporosis of the low-risk, medium-risk, and high-risk category was 23.4% (1353/5782), 44.7% (1418/3170), and 70.0% (2256/3223), respectively.

## Discussion

Several osteoporosis risk assessment tools based on clinical risk factors are available to predict osteoporosis [9–16]. The OSTA index developed by Koh et al. [9], is a simple tool to predict postmenopausal women with osteoporosis and had been validated in the subsequent investigations [18–23].

In current investigation, we aimed at development of a simple diagnostic tool for identifying subjects with risk of osteoporosis in Taiwan postmenopausal women and analyzing the diagnostic performance of OSTAi and NOF 2013 in our cohort. With linear regression model and item reduction step by step as developed by Koh et al., we found that age and BW were the 2 major factors in development of the OSTAi index. Aging was directly related to the presence of osteoporosis, whereas increase of weight was inversely related. Though the factors incorporated in current investigation are different from the original model, the algorithm for calculating OSTAi value is exactly the same as the original model.

Prevalence of osteoporosis for high-risk group by OSTA risk category in several Asian studies, including multi-country sample of Asian women[9], Korea[19], Filipinos[21], China[23] were 61%, 64%, 51.3%, and 44%, respectively. As the cutoff index values of each study were arbitrary, to compare the prevalence of osteoporosis of each country/population in each category seems not feasible. However; it seems that OSTAi, based on selected cut-off index value, is an appropriate tool to identify high-risk subjects with osteoporosis in Taiwan postmenopausal women for DXA referral as more than half of those women below cut-off index value had osteoporosis. Hence, DXA testing should be recommended to confirm the diagnosis of osteoporosis for those women with OSTAi index value <-1.

In addition, it also demonstrated that the selected cut-off value (= -1) provided high sensitivity (72.6–89.6%) and fair specificity (51.1–62.0%) in selecting subjects with osteoporosis at different skeletal sites. The results were similar to a previous validation study conducted in 1201 postmenopausal Han Chinese women [23]. In that study, it disclosed sensitivity (74–83%) and specificity values (63–68%) of the OSTA index (an cutoff value of -1) for the diagnosis of osteoporosis (T-score ≤ − 2.5 SD) using BMD measurements at the femoral neck, total hip, and lumbar spine.

Several previous studies defined osteoporosis by a T-score ≤ −2.5 at the femoral neck for establishment of OSTA [9, 18, 19, 21]. However, based on BMD measured at different skeletal sites, our data showed that the accuracy for OSTAi was somewhat diverse. Using a cutoff value of -1, the accuracy of OSTAi for diagnosis of osteoporosis in the lumbar spine, femoral neck, total hip, and at any site were 0.62, 0.63, 0.55, and 0.67, respectively. Furthermore, the optimal OSTAi cutoff may vary based on different skeletal sites. In addition, it was well known that osteoporosis at any site can predict future fracture risk at other site [32]. Therefore, as the study by Yang et al. [23] and in Saraví FD research [33], we developed OSTAi for identifying possible osteoporosis according to T-score ≤ -2.5 at any site, instead of the femoral neck in other studies [9, 18, 19, 21].

In terms of performance, OSTAi and NOF 2013 were analyzed. It disclosed that both PPV and NPV of OSTAi, at the cutoff value of -1, (57.5% and 76.6%) were higher than those of NOF 2013 (50.8% and 75.4%) (Table 4). Although there was higher sensitivity (78.3%) of NOF 2013 than of OSTAi (73.1%), the specificity of OSTAi (62.0%) was higher than NOF 2013 (46.7%) and the AUC of OSTAi (0.739, CI. 95%, 0.728–0.749, P<0.001) was higher than that of NOF 2013 (0.618, CI. 95%, 0.606–0.630, P<0.001). Compared to NOF 2013, it seems that OSTAi provides a better prediction for Taiwanese postmenopausal women at risk of osteoporosis for DXA referral.

One study in Korea [27] also compared the performance of OSTA with NOF 1999. In that study, women aged 40 years or older (N = 837) without diseases affecting BMD were evaluated with information about risk factors for fracture and the results of BMD. The AUC of the OSTA and NOF 1999 were 0.802 and 0.709 respectively. The results also indicated that NOF 1999 did not perform better in osteoporosis risk prediction compared with the OSTA in detecting osteoporosis in Korean perimenopausal or postmenopausal women [27].

Despite that the OSTA for Taiwanese young adult women (aged 30–49 years) has been established by Chang SF et al. However, it had not been validated for postmenopausal women in Taiwan [34]. To our knowledge, current investigation is the first nationwide survey of osteoporosis with a large sample size in Taiwan that may represent the country wide population of Taiwan postmenopausal women. The strength of our study is the greater participants than other survey, which can produce more reliable tool. In our study, BMD measurements were performed by the same certified technician and the same DXA machine throughout the study that would avoid the potential inter-modality and inter-operator variation. One of the inevitable limitations of previous investigations was the operation by different technicians or use of different brands of densitometry equipment. [9, 13, 16, 20, 27] that would potentially yield non-comparable BMD data. In addition, we incorporated the risk factors of fragility fracture in FRAX tool for the development of OSTAi in this model that had not been utilized to establish the OSTA model.

Our study has several limitations. First, samples were not random populations and prevalence of osteoporosis might be overestimated. For example, the prevalence of RA and glucocorticoid uses comprised 7.4% and 6.8% that are higher than general population in Taiwan [30, 35]. That is due to certain proportion of subjects in our sample were referred by health-care provider for osteoporosis evaluations. These participants might have higher rate of osteoporosis. However, even after these participants were excluded from analysis, the sensitivity/specificity of OSTAi was similar when factors of RA and glucocoticoid use were considered. Moreover, the prevalence of osteoporosis in this study was similar to one previous investigation regarding the prevalence in Taiwanese women aged 50 years and older, which the prevalence rate of osteoporosis at any site were 38.3% [36]. It has been demonstrated that aging and lower body weight can predict future fracture risk [37]. As current investigation is a cross-sectional study, whether the OSTAi index can predict the future fracture risk for postmenopausal women in Taiwan needs further investigation.

In conclusion, the OSTAi index not only is a simple tool to select Taiwan postmenopausal women with osteoporosis. And it also demonstrates adequate sensitivity/specificity and PPV/NPV. In addition, the OSTAi index is a simpler and better tool for DXA referral than NOF 2013 recommendations.

## Supporting Information

S1 File Data and measurements of BMD (T-scores) in enrolled participants. All the information was anonymized.
(XLSX)
